# Chronic exposure to inorganic arsenic and fluoride induces redox imbalance, inhibits the transsulfuration pathway, and alters glutamate receptor expression in the brain, resulting in memory impairment in adult male mouse offspring

**DOI:** 10.1007/s00204-023-03556-7

**Published:** 2023-07-23

**Authors:** Wendy L. González-Alfonso, Petrosyan Pavel, Hernández-Mercado Karina, Luz M. Del Razo, Luz C. Sanchez-Peña, Angélica Zepeda, María E. Gonsebatt

**Affiliations:** 1 Departamento de Medicina Genómica, Instituto de Investigaciones Biomédicas, Universidad Nacional Autónoma de México, A. P. 70-228, Ciudad Universitaria, 04510 Mexico, CDMX México; 2 Departamento de Toxicología, Centro de Investigación Y Estudios Avanzados, Mexico, DF Mexico

**Keywords:** Arsenic, Fluoride, xCT, Transsulfuration pathway, Hydrogen sulfide, Glutamate receptor, Glutamate disposal

## Abstract

Exposure to toxic elements in drinking water, such as arsenic (As) and fluoride (F), starts at gestation and has been associated with memory and learning deficits in children. Studies in which rodents underwent mechanistic single exposure to As or F showed that the neurotoxic effects are associated with their capacity to disrupt redox balance, mainly by diminishing glutathione (GSH) levels, altering glutamate disposal, and altering glutamate receptor expression, which disrupts synaptic transmission. Elevated levels of As and F are common in groundwater worldwide. To explore the neurotoxicity of chronic exposure to As and F in drinking water, pregnant CD-1 mice were exposed to 2 mg/L As (sodium arsenite) and 25 mg/L F (sodium fluoride) alone or in combination. The male litter continued to receive exposure up to 30 or 90 days after birth. The effects of chronic exposure on GSH levels, transsulfuration pathway enzymatic activity, expression of cysteine/cystine transporters, glutamate transporters, and ionotropic glutamate receptor subunits as well as behavioral performance in the object recognition memory task were assessed. Combined exposure resulted in a significant reduction in GSH levels in the cortex and hippocampus at different times, decreased transsulfuration pathway enzyme activity, as well as diminished xCT protein expression. Altered glutamate receptor expression in the cortex and hippocampus and decreased transaminase enzyme activity were observed. These molecular alterations were associated with memory impairment in the object recognition task, which relies on these brain regions.

**Supplementary Information:**

The online version contains supplementary material available at 10.1007/s00204-023-03556-7.

## Introduction

Fluoride (F) and arsenic (As) are the most serious contaminants in drinking water worldwide and are of health concern, since approximately over one-third of the world´s population depends on the ground water supply (Schmoll et al. 2006). Chronic exposure to As and F by drinking water has been linked to toxic effects in several organs and to neurodevelopmental alterations in children (Tyler and Allan 2014, Agalokova and Nadei 2020). As and F can cross the placenta and blood–brain barrier and can incorporate into the central nervous system (CNS). As is a metalloid element with high affinity for the SH- group, while F has strong electronegativity with high affinity for OH- and NH-, which results in the alteration of protein assembly and other molecular interactions. Alteration in glutathione (GSH) levels is the main redox perturbation associated with As or F exposure (Chouhan and Flora 2010). However, although As + F are frequently present in groundwater worldwide (Schmoll et al. 2006), the effects of the combination of these elements in drinking water have been poorly explored (Mondal and Chattopadhyay 2020). l-γ-Glutamyl-l-cysteinyl-glycine (GSH) constitutes the major antioxidant defense in the brain, and its synthesis is limited by cysteine availability. Brain cells take up cystine or cysteine through facilitated transport and synthesize cysteine from methionine through the transsulfuration pathway (Lu 2013). The transsulfuration pathway involves the participation of enzymatic steps carried out by cystathionine β-synthase (CBS) and cystathionine γ-lyase (CSE) (Paul et al. 2018). Cysteine in the extracellular space is mostly oxidized as cystine, and its uptake by glial cells is mediated mainly by the antiporter xCT in exchange for glutamate. Neuron cysteine uptake is mediated by EAAT3 (EACC1), which is part of the excitatory amino acid transporter (EAAT) family (Valdovinos-Flores and Gonsebatt 2012). GSH homeostasis in the brain is tightly linked to glutamatergic synaptic transmission, since GSH can also serve as a reservoir of neuronal glutamate. Additionally, xCT and EAAT3 participate in the uptake of Cys and may also modulate extracellular glutamate levels.

During glutamatergic transmission, vesicular glutamate release activates ionotropic glutamate receptors in the postsynaptic terminal. The most abundant receptors include α-amino-3-hydroxy-5-methyl-4-isoxazolepropionic acid (AMPA) and N-methyl-D-aspartate (NMDA) receptors, which depolarize the postsynaptic terminal upon activation to propagate the nervous impulse. The signal ends when glutamate is removed from the synaptic cleft (Hassel and Dingledine 2012). Glutamate homeostasis is essential for proper signal transmission and depends on coordinated glutamate recycling and metabolism between neurons and astrocytes. Vesicular glutamate originates from the glutamate–glutamine shuttle and from de novo synthesis mainly by aspartate aminotransferase (AST) and alanine amine transferase (ALT). Moreover, glutamate recapture is mediated by EAAT transporter family members that are expressed in high levels on astrocytes but also in the synaptic terminals (Andersen et al. 2021).

We have previously explored the neurotoxic impact of chronic As exposure starting at gestation and observed disrupted glutamate disposition in the cortex and hippocampus. Increased expression of the cystine/glutamate xCT transporter was linked to increased levels of extracellular glutamate in the hippocampus and altered expression of NMDA and AMPA receptors (Ramos-Chavez et al. 2015; Nelson-Mora et al. 2018). In the case of F exposure during gestation and lactation, different groups have reported reduced activity and expression of antioxidant components in the cortex and hippocampus of rat and mouse offspring. Additionally, they observed reduced activity of brain transaminase involved in glutamate synthesis and altered expression of glutamate receptors, associated with memory impairment (Sun et al. 2018; Bartos et al. 2022). In Mexico, levels as high as 0.650 mg/L and 23.4 mg/L for As and F, respectively, have been observed in some locations. As and F are present in a ratio of approximately 1:20–1:50 (Alfaro de la Torre et al. 2018). Rodent models show a resistance to As or F toxicity of five-to-tenfold compared to that of humans (Vahter 1999; Council 2006). In this work, we explored the effects of exposure to low and relevant doses of these toxic elements considering the mouse model resistance, the As:F ratio, and average levels in nature. To explore the impact of As + F in drinking water on the cortex and hippocampus, CD1 male mice were chronically exposed at gestation to 2 mg/L sodium arsenite and 25 mg/L sodium fluoride in drinking water. Glutathione levels, transsulfuration pathway enzyme activities, and the expression of cystine/glutamate and cysteine transporters as well as NMDA and AMPA receptor proteins were explored in the cortex and hippocampus at postnatal days 30 and 90 (P30 and P90, respectively). Additionally, the expressions of PSD95 and synaptophysin were determined to investigate the general postsynaptic protein distribution and arrangement and vesicular density, respectively. Behavioral tests to evaluate learning and memory were performed at P90.

## Materials and methods

### Chemicals and antibodies

All chemicals were purchased from Sigma-Aldrich (St Louis, MO, USA) unless otherwise indicated. Methylarsonic acid (MMAV) disodium salt (99% pure) was obtained from Chem Service (West Chester, PA, USA). Sodium borohydride was obtained from EM Science (Gibbstown, NJ, USA). For Western blots, primary rabbit antibodies against xCT (ab37185), EAAT3 (ab124802), GLAST (ab416), GLT1 (ab41621), NR2B (ab65783), GluA2 (ab133477), Synaptophysin (SY38, ab8049), CBS (ab135626), and CSE (ab151769) were obtained from Abcam (Cambridge, MA, USA). Anti-LAT1 (sc-134994) and PSD95 (7E3, sc-32290) were purchased from Santa Cruz Biotechnology (Santa Cruz, CA, USA). Rabbit anti-NR2A (AB1555P) and anti-GluA1 (AB1504) antibodies were purchased from Millipore (Bedford, MA, USA). Primary antibody against SLC1A4 (8442 s) and secondary goat anti-rabbit antibodies were obtained from Cell Signaling Technology (Danvers, MA, USA). Secondary anti-mouse IgG antibody was purchased from Invitrogen (Waltham, MA, USA).

### Animals and treatment

Eight- to ten-week-old CD-1 mice were obtained from the Animal Care Facility at the Instituto de Investigaciones Biomédicas, UNAM and were maintained at 23–25 ℃ under a 12 h light/dark cycle and a relative humidity of 50–60%. Animals were given free access to food (Harlan 2018S Diet; Harlan, Indianapolis, IN, USA) and water. The experimental protocol was approved by the Institutional Animal Care and Use Committee of the Instituto de Investigaciones Biomedicas, UNAM. Virgin female mice were paired with proven breeder males, and the next day, each female was explored for the presence of a mating plug. Pregnant mice (*n* = 40) were randomly divided into four groups. The first group (control group) received iAs/F-free drinking water, the second group (As group) received 2 mg/L As daily as sodium arsenite in drinking water, the third group (F group) received 25 mg/L F daily as sodium fluoride in drinking water, and the fourth group (combination As + F group) received both elements in the above-mentioned concentrations in drinking water. Solutions were prepared freshly daily in deionized water to avoid oxidation of As. Animal weight was determined weekly. At the end of the lactation period (postnatal day 21), mice were separated by sex. Male litters continued with the exposure protocols until P30 or P90. At these times, mice were euthanized, and the cortex as well as the hippocampus were dissected, immediately frozen, and stored at − 70 ℃ until processed or used freshly for some experiments.

Animals were handled following the ‘‘Principles of Laboratory Animal Care’’ guidelines (NIH publication #85-23, revised 1985) and ‘‘Especificaciones técnicas para la producción, cuidado y uso de los animales de laboratorio (Clave NOM-062-ZOO-1999)’’ of the ‘‘Norma Oficial Mexicana de la Secretaría de Agricultura, Ganadería, Desarrollo Rural, Pesca y Alimentación (SAGARPA)’’ (published in August 2001).

### Arsenic level determination

The levels of As species were determined in the cortex and hippocampus by hydride-generation atomic absorption spectrometry using cryotrapping (HG-CT-AAS) as previously described by Hernández-Zavala et al. (2008). First, tissue sample homogenates were prepared on ice with deionized water as described by Currier et al. (2011). Independent calibration graphs of the inorganic and methylated arsenic species were used for quantification.

### Fluoride level determination

The levels of F were determined in pooled samples of four mouse cortex per group, combining the isothermal distillation technique (Rigalli and Puche 2007) with a potentiometric method using an ion-selective electrode (Orion 9609BNWP, Thermo Fisher Scientific Inc., USA). A 1:1 mixture of the sample and a total ionic strength adjustment buffer (TISAB) were made. The concentration of F was determined by interpolating the results on a calibration plot. Urine fluoride reference materials PC-U-F2102 (0.413 μg/mL), PC-U-F2103 (7.31 μg/mL), and PC-U-F2104 (4.25 μg/mL) from the Centre de Toxicologie du Québec (Institut National de Santé Publique du Québec [INSPQ]) were used as quality control.

### GSH level determination

Reduced GSH levels were measured in fresh tissue preparations of the cortex and hippocampus using a microplate-adapted fluorometric o-phthalaldehyde (OPA) method (Silva-Adaya et al. 2020). The method is based on the conjugation of reduced GSH with o-phthalaldehyde (OPA), forming a stable and fluorescent isoindole derivate (Senft et al. 2000). GSH levels were determined by fluorescence with 365 nm/430 nm (excitation/emission) filters in a DTX 800/880 Multimode Detector (Beckman Coulter, Fullerton, CA, USA).

### Transsulfuration pathway activity

CBS and CSE activity was determined by their ability to produce H_2_S (Silva-Adaya et al. 2020). H_2_S production was measured through the formation of PbS spots (brown dot spots), which resulted from the reaction of diffused H_2_S with PbAc embedded in the filter paper placed over the plate surface. The assay was run in a 96-well plate with at a final volume of 150 μl containing 300 µg of protein from homogenated cortex or hippocampus and reaction solution (final concentration of 10 mM L-Cys, 1 mM PLP in PBS1X) per well. Liver samples from untreated animals were used as positive controls. Dot spots were quantified using ImageJ software version 1.46r software (US National Institutes of Health, Bethesda, MD, USA).

### Western blotting

Total protein preparations from the cortex and the hippocampus were used as the source of protein for the Western blot studies. Briefly, 20 mg of tissue was homogenized in RIPA buffer (Tris HCl 50 mM, pH 7.55, NaCl 150 mM, EDTA 2 mM, EGTA 1 mM, DTT 1 mM, NaPPi 2.5 mM, Triton X-100 1%, sodium deoxycholate 0.1%, glycerol 2-P 1 mM, Na_2_VO_4_ 1 mM, PMSF 1 mM, and 10 mg/mL aprotinin/leupeptin) and centrifuged at 14,000×g for 15 min at 4 ℃. The supernatant was collected and stored at − 80 ℃ for further analysis. The total protein concentration was determined by the microtiter Bradford technique (Bio-Rad, CA, USA) using a standard graph of albumin. *R*^2^ > 0.98. For transporter and enzyme expression analysis, 30 μg of protein was loaded in a 10% SDS-acrylamide gel and transferred into nitrocellulose (Bio-Rad Laboratories, Germany). For receptor expression analysis, 50 μg of protein was loaded in 8% SDS-acrylamide gel and transferred into PVDF membranes (Bio-Rad Laboratories, Germany). The membranes were blocked with TBS containing 5% Blotto and 0.1% Tween-20 and incubated with the respective primary antibodies at 4 °C overnight. As a loading control, total protein Ponceau staining was used. Protein bands were visualized after incubation with the appropriate HRP-linked secondary antibodies using the Amersham TM ECL TM Advance Western blotting Detection Kit (GE Life Sciences, RPN2232). Images were captured using Li-Cor and analyzed using ImageJ software version 1.46r software (U.S. National Institutes of Health, Bethesda, Maryland, USA). Protein expression levels were analyzed using ImageJ (vs. 1.49) and normalizing each band with respect to the loading control (Ponceau Staining). Protein contents were finally compared and represented with respect to the experimental control.

### Brain transaminase activities

Transaminase activity was determined as a marker of de novo synthesis of glutamate by a modified procedure (Reitman and Frankel 1957) using dinitrophenyl-hydrazine (DNPH). Briefly, 40 μg of protein was incubated with 100 μL of ALT substrate (2 mM α keto-glutarate + 0.2 M alanine in phosphate buffer pH 7.4) or AST substrate (2 mM α keto-glutarate + 0.2 M aspartate in phosphate buffer pH 7.4) at 37 °C for 30 min or 60 min, respectively. Subsequently, 100 μL of 1 mM DNPH in 1 N HCl was added to the mixture and incubated at RT for 20 min. Finally, 1 mL of NaOH1M was added and incubated for 10 min at RT. The absorbance at 505 nm was measured. Sodium pyruvate (2 mM) was used as a standard, and enzymatic activity was expressed as U/L using the following equations: ${\text{EA }}\left( {{\text{AST}}} \right) = \frac{{{\text{umol}}\left( {{\text{60min}}} \right) - {\text{umol}}\left( {0{\text{min}}} \right)}}{{60{\text{min}}\,\, \times \,\,0.00001{\text{L}}}}$, ${\text{EA }}\left( {{\text{ALT}}} \right) = \frac{{{\text{umol}}\left( {60{\text{min}}} \right) - {\text{umol}}\left( {0{\text{min}}} \right)}}{{30{\text{min}}\, \times \,\,0.00001L}}$, respectively.

### Behavioral tasks

Behavioral studies were performed on P83–P90 following previously described protocols (Moreno-Castilla et al. 2017). For the open-field test, as well as for the object and place recognition tests, we used a white acrylic closed square arena (40 × 40 × 25 cm) in which the floor was covered with sawdust. The arena was placed in a dim-light illuminated room and had an internal visual clue consisting of a rectangular black and white striped plasticized cardboard (38 × 5 cm) glued to one of the walls (see Supplementary Fig. 1). The objects used in the recognition tasks were two identical pyramids of Lego blocks named “A1” and “A2” (4.8 cm length × 3.0 cm width × 4.0 cm height, built in red, blue, gray, and yellow) and a different object, a tower of Lego blocks referred to as “novel object” (NO, 3.2 cm length × 1.6 cm width × 4.5 cm height, built in green, red, yellow, and white) (see Supplementary Fig. 1). The objects were thoroughly cleaned with 70% ethanol, and sawdust was stirred after each trial to prevent olfactory cues. The animals were transported to the experimental room 30 min before the beginning of each session. All behavioral sessions were video-recorded for further analysis. One of the two experimenters remained blinded to the treatment of each animal. The blind experimenter performed the calculations to determine the exploration index in the acquisition and test phases of the recognition tasks (see below).

#### Habituation to the arena and open-field test

Each mouse was handled for 10 min on 3 consecutive days and was then habituated to the squared arena without any objects for 10 min a day for 3 days before the onset of the memory tasks. The first day of habituation was used to evaluate the general motor performance and anxiety-related behaviors through an open-field analysis. This consisted of the measurement of the total traveled distance, the percentage of time spent in the center or peripheral zones of the arena (see Supplementary Fig. 1), and the rearing frequency. General behavior recording and further observational analysis were performed using EthoVision XT software (11.5.126, Wageningen, The Netherlands).

#### Novel object recognition task (NOR)

On day 4 (after 3 days of habituation), each mouse was introduced to the arena and allowed to explore two identical objects (A1 and A2) for 10 min for the acquisition phase. Objects were fixed on the floor close to one of the corners of the arena (8 cm away from each of the two adjacent walls, see Supplementary Fig. 1) with double-sided tape to prevent displacement by the animals. On day 5, each mouse was introduced to the arena where one familiar object (FO, A1) and one novel object (NO) were placed. The objects were placed in the same positions as in the acquisition phase (Supplementary Fig. 1), and subjects could freely explore the objects for 10 min. An exploration index for the novel object, reflecting object memory, was further obtained.

#### Location of object recognition task (LOR)

On day 6, each mouse was placed in the arena and could freely explore the same objects used on day 5 for 10 min for the acquisition phase (see Supplementary Fig. 1). The objects were placed in the same position as on day 5 and are referred to as location 1 (L1) and location 2 (L2) in Fig. 5B. On day 7, the object located in L1 was placed in the same position as in the acquisition phase (“familiar location”, FL), and the object located in L2 was placed in a “novel location” (NL). Mice could freely explore the objects for 10 min (see Supplementary Fig. 1). An exploration index for the object in the novel location, reflecting place memory, was further obtained.

The results were expressed as an exploration index (EI) that was calculated as the time spent exploring (in seconds) a specific object over the total exploration time (both objects) IE = [object 1/(object 1 + object 2)]. An EI equal to 0.5 indicates no preference for any object or location, while an EI higher than 0.5 is considered a preference for an object or a location. Exploration was considered as pointing the nose to an object at 2 cm or less and touching it with the nose. Turning around, sitting on, climbing, or gnawing the objects was not considered exploratory behavior.

### Data analysis

The data are expressed as the mean ± standard error of the mean. The number of animals tested is indicated in each case. One-way analysis of variance (ANOVA) tests were used to assess statistical significance followed by post hoc tests, as indicated in the corresponding figures. A *p* < 0.05 was considered statistically significant in all cases.

## Results

Exposed pregnant females did not show significant differences from the controls with respect to weight gain, food or water consumption, or litter size or weight at birth (data not shown). Pup weight gain was also similar during and after lactation. To investigate the effects of exposure in the offspring, we determined the content of arsenical species (inorganic and methylated forms) in the cortex and hippocampus in mice at P30 and P90. In most mammals, including humans, inorganic As is reduced and methylated into mono-methylated and di-methylated trivalent and pentavalent forms and conjugated with GSH in most tissues, including the brain (Sánchez-Peña et al. 2010). Total As levels in the cortex were higher than those in the hippocampus. The levels of methylated As species in the mouse cortex of animals exposed to As were significantly higher than those exposed to As + F at P30 (As: 8.64 ± 1.54 vs. As + F: 3.76 ± 2.21 ng/g tissue), while this difference was not observed at P90 (As: 4.37 ± 0.67 vs. As + F: 5.47 ± 1.69 ng/g tissue). The levels of methylated As species in the hippocampus were not significantly different at P30 or P90 (As: 3.81 ± 0.39 vs. As + F: 3.35 ± 1.95 ng/g tissue; As: 1.65 ± 1.27 vs. As + F: 1.92 ± 1.05 ng/g tissue, respectively). During lactation, animals are not exposed to As due to the inability of As to accumulate in maternal milk. The higher levels observed at P30 compared with P90 could be a boost in accumulation after the restart of exposure after ending the lactation period (Hughes et al. 2003). In contrast, we observed similar levels of F in the cortex among groups. The F concentrations determined in the P90 pooled cortex samples were 0.019 µg/g in control, 0.023 µg/g in As exposed group, 0.019 µg/g in F-exposed group, and 0.023 µg/g in the combined AS + F group. F concentration in soft tissues such as the brain is usually low and also dose- and time-dependent (Inkielewicz and Krechniak, 2003). A few studies have reported its accumulation in brain using higher doses and longer periods of exposure than those used here (Inkielewicz and Krechniak, 2003; Sharma et al. 2022).

### GSH levels in the cortex and hippocampus are affected by combined exposure to As and F

**Fig. 1:**
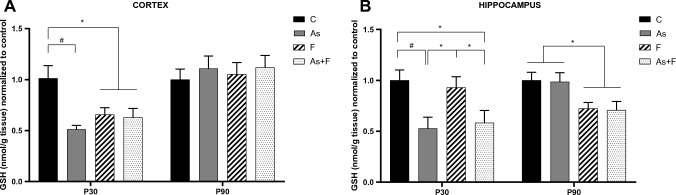
Effect of As and F exposure on GSH. GSH levels in the mouse cortex (**A**) and hippocampus (**B**) at P30 and P90. Bars show means ± SEMs, normalized against controls. Data were analyzed by two-way ANOVA followed by Tukey’s post hoc analysis. *P < 0.05, #*P* < 0.01, *n* = 7

At P30, the levels of reduced GSH were significantly decreased in the cortex of animals exposed to As, F, or As + F (Fig. 1A), while at P90, the level of GSH in all groups was similar to that of controls, suggesting that GSH synthesis increased in the exposed groups after P30. In contrast, in the hippocampus, GSH levels were significantly lower at P30 in the As and As + F groups, while mice exposed to F showed levels similar to control. At P90, only mice receiving F or As + F showed significantly lower levels of GSH (Fig. 1B). These results suggest that the hippocampus shows evidence of continued oxidative stress by As + F due to the significantly lower levels of reduced GSH. Additionally, the combination As + F showed different impacts on the GSH system depending on age and brain region.

### CBS and CSE activity inhibition is associated with lower GSH levels in As- and F-exposed mice

**Fig. 2:**
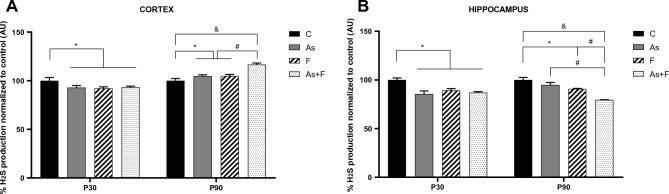
H_2_S production in the mouse cortex (**A**) and hippocampus (**B**) at P30 and P90. Bars show means ± SEMs of the densitometric measurement of PbS spots formed, normalized against controls. Data were analyzed by two-way ANOVA followed by Tukey’s post hoc analysis. *P < 0.05, #*P* < 0.01, & *P* < 0.0001, *n* = 5 (a) *n* = 7 (b)

Cysteine bioavailability is a limiting factor for GSH synthesis and the maintenance of adequate levels of GSH in cells. We investigated the expression and activity of CBS and CSE. CBS and CSE protein levels in the cortex or in the hippocampus were not altered at P30 or P90 (Supplementary Fig. 2A, B). However, inhibition of the enzymatic activity measured as H_2_S production was observed in groups exposed to As and F. In the cortex, activity at P30 was initially reduced, and then, the activity of the enzymes at P90 increased, especially in the As + F group; this observation could be linked to the decrease (P30) and later (P90) increase in GSH levels, respectively (Fig. 1A). In contrast, H_2_S production in the hippocampus was reduced in all exposed groups compared to the control at P30 and continued to be inhibited or reduced in the F- and As + F-exposed groups at P90 (Fig. 2A, B, Supplementary 2C), which coincides with the lower levels of GSH observed at P90.

### Expression of cystine/glutamate, cysteine, and glutamate transporters

**Fig. 3:**
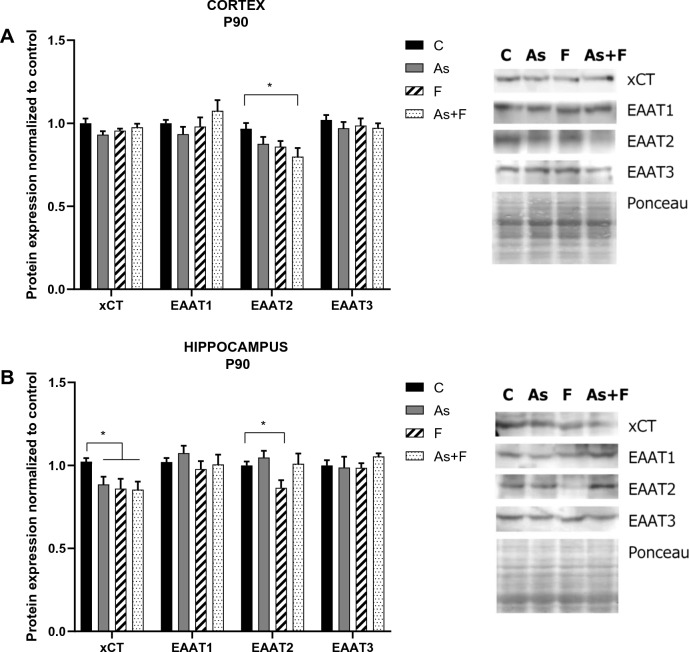
Cystine/glutamate and glutamate transporter expression in the mouse cortex (**A**) and hippocampus (**B**) at P90. Bars show means ± SEMs of the densitometric determination of Western blot images normalized against total protein stain as loading control and expressed respect to controls. Data were analyzed by two-way ANOVA followed by Tukey’s post hoc analysis. *P < 0.05, *n* = 6

A significant reduction in EAAT2 protein levels (glutamate importer) was observed in the cortex at P90 in the As + F-exposed group (Fig. 3A). Simultaneously, in the hippocampus, we observed an important reduction in the level of xCT at the mRNA and protein levels at P90 in the group exposed to As and/or F compared to the control group and of EAAT2 in the mice exposed to F (Fig. 3B).

### Modulation of ionotropic glutamate receptor expression and glutamate synthesis

**Fig. 4:**
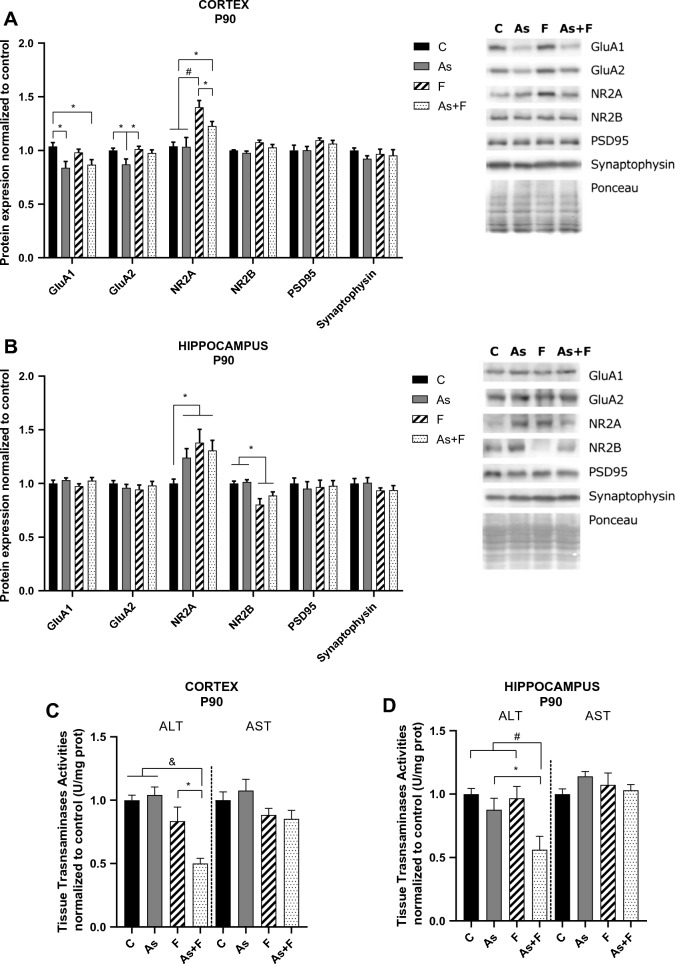
Glutamate ionotropic receptor subunit, PSD95, and synaptophysin protein expression in mouse cortex (**A**) and hippocampus (**B**) on P90. Bars represent means ± SEMs of densitometric determination of Western blot images normalized against total protein stain as loading control and expressed respect to controls. Data were analyzed by two-way ANOVA followed by Tukey’s post hoc analysis (*n* = 6). Brain tissue transaminase activity at P90 in the cortex and hippocampus (C-D). Bars represent means ± SEMs. Data were analyzed by two-way ANOVA followed by Tukey’s post hoc analysis (*n* = 7). *P < 0.05, #*P* < 0.01, & *P *< 0.0001

Alterations in glutamatergic transmission have been associated with neurological impairment after As or F exposure (Tyler and Allan 2014; Agalakova and Nadei 2020). Here, we observed crucial differences between glutamate receptor subunit expressions in exposed animals at P90, the time when the behavioral tasks were performed. In the cortex, As exposure reduced the expression levels of GluA1 and GluA2, but no changes were observed in NR2A or NR2B subunits. F exposure increased the protein levels of NR2A without altering AMPAR expression. In contrast, As + F exposure reduced the protein levels of GluA1 and increased the levels of NR2A (Fig. 4A).

In the hippocampus, no changes were observed in AMPAR at P90, but an increase in NR2A and a decrease in NR2B in the F and As + F groups were observed at this same time point (Fig. 4B). In addition, we tested the expression of PSD95 and synaptophysin in the cortex and hippocampus as biomarkers of general postsynaptic protein distribution and arrangement and vesicular density, respectively. We did not observe significant alterations in any of the protein contents, suggesting that glutamate synaptic transmission is more discretely modulated at the doses tested compared to the ultrastructural modification of the synapse observed in other models exposed to higher doses (Luo et al. 2009; Huo et al. 2015).

### De novo glutamate synthesis

ALT and AST are responsible for de novo glutamate synthesis in the brain. A significant reduction in ALT activity was observed in the cortex and hippocampus at P90 in the As + F group. These results suggest an important alteration in glutamate availability in the brain (Fig. 4C, D).

### Memory impairment after combined exposure to As and F

**Fig. 5:**
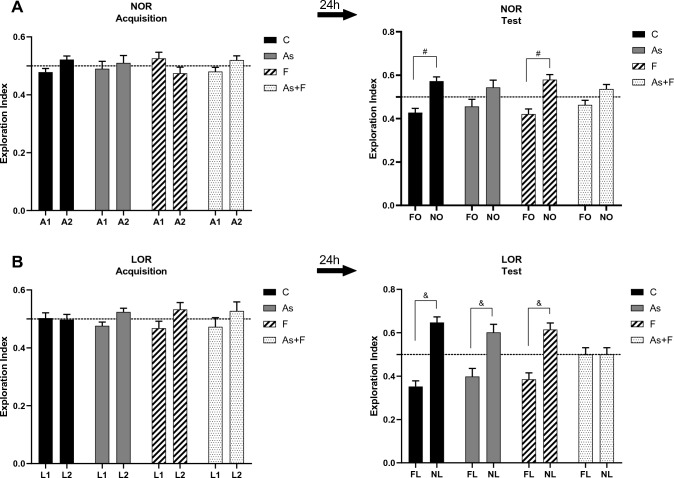
Memory impairment in the novel object recognition (NOR) and location of object recognition (LOR) tasks at P90 in animals exposed to As and As + F. **A**) Exploration index (EI) during object acquisition (left graph) and the test phase of NOR (right graph). During acquisition, animals explored the two identical objects for a similar amount of time, showing no difference in the EI. During the test phase (right panel), animals exposed to As and As + F did not display a significant EI for the novel object (NO). B) EI during the acquisition (left graph) and test phases (right graph) of LOR. During acquisition, animals explored the objects in the known locations for a similar amount of time, showing no difference in the EI. During the test phase (right panel), all groups spent more time exploring the novel location except for the As + F group. EI equal to 0.5 indicates no preference for any particular object/location, while a value higher than 0.5 indicates a preference for an object/location. A1 and A2 are identical objects; NO is a novel object; FL indicates familiar location; NL indicates novel location. Mean ± SEM. *n* = 6 (control, As + F), *n* = 8 (As), *n* = 7 (F) two-way ANOVA, post hoc test: Tukey’s test. #*P* < 0.01, & *P* < 0.0001

Memory performance was assessed using the following recognition tasks: the novel object recognition (NOR) and the location object recognition (LOR) tests to establish a possible association between the observed molecular alterations and behavioral impairments. NOR has a meaningful cortex participation, while the LOR has a critical spatial memory component that involves the hippocampus (Vogel-Ciernia and Wood 2014). The analysis of the open-field test performed prior to the NOR task did not show significant motility, alteration in exploration behavior, or anxiety-like behavior induced by As or F treatment (Supplementary Fig. 3), which was important for the further interpretation of memory performance (Vogel-Ciernia and Wood 2014). During the acquisition phase in both tasks, NOR and LOR, no preference was observed for the two identical objects, as expected (Fig. 5A and B). In the test phase of NOR, mice exposed to As and to As + F could not discriminate the novel object (NO) in contrast to the control group, suggesting a memory impairment related to the cortex region (Fig. 5A). In the LOR test phase, we also observed an impairment in the ability to discriminate the novel location (NL) in the group exposed to As + F (Fig. 5B, p < 0.05), which suggests an impairment in spatial memory.

## Discussion

Groundwater contamination with As and F is common in arid and semiarid regions from Latin America, Asia, and Africa, affecting over 300 million people worldwide. Unfortunately, overexploitation of groundwater increases the concentration of toxic elements (Schmoll et al. 2006; Falkenmark 2009). In many of these regions, as in México, populations have been exposed for generations to groundwater with elevated levels of As and F (Limón-Pacheco 2018). This condition has caused detrimental effects on their quality of life due to the higher prevalence of cardiovascular, renal, hepatic, reproductive, and neurological diseases (Mondal and Chattopadhyay 2020). Learning and memory impairments have been documented in children exposed to As or F worldwide (Tyler and Allan 2014; Agalakova and Nadei 2020). Using a mouse model, we have previously reported that exposure to inorganic As in drinking water starting at gestation alters the homeostasis of GSH and disrupts the availability of glutamate in the hippocampus, which was associated with altered ionotropic glutamate receptor expression, and to impaired memory (Ramos-Chavez et al. 2015; Nelson-Mora et al. 2018). On the other hand, F also induces an imbalance in redox homeostasis in the brain and affects glutamatergic transmission, as reviewed by Agalakova and Nadei (2020).

In this work, we further show that As + F exposure combined in drinking water led to an important reduction in GSH levels at P30 and P90 in the cortex and hippocampus (Fig. 1). The decrease in GSH levels was shown to occur along a reduction in CBS and CSE activities and xCT protein expression in the hippocampus at P90. In the cortex at P90, GSH levels were recovered in association with an induction in CBS and CSE activity (Figs. 2 and 3). Significantly lower levels of GSH, xCT downregulation, and transsulfuration inhibition could lead to ferroptosis in the hippocampus (Stockwell et al. 2020), although additional experiments are needed to further strengthen these suggestions.

On the other hand, the modulation in the transsulfuration pathway could be due to allosteric regulation. S-adenosylmethionine (SAM) is an allosteric activator of CBS (Prudova et al. 2006). However, SAM levels could be diminished due to As methylation (Sánchez-Peña et al. 2010) and by F-induced inhibition of methionine adenosyl-transferase (al-Khafaji et al. 1998). Determining the SAM levels in this model would clarify whether the transsulfuration pathway inhibition observed in the hippocampus and cortex (P30) is mediated by SAM bioavailability alteration.

On the contrary, the activity of the pathway could be induced under toxic conditions. The increase in transsulfuration observed in the cortex at P90 (Fig. 2A), especially in the group exposed to the combination of As + F, could be associated with a higher accumulation of both elements combined in that and, therefore, a synergistic induction of adaptive response to overcome redox imbalance.

As mentioned before, changes in GSH levels could lead to altered glutamate homeostasis. The As + F exposure group showed greater effects on transporters and enzymes that regulate glutamate levels, including the inhibition of transaminase activity and the decreased expression of EAAT2 and xCT (Fig. 4C and Fig. 3, respectively). EAAT2 is the major member of the EAAT family present in the forebrain and is found primarily in astrocytes and neurons. EAAT2 is responsible for the most glutamate uptake; therefore, downregulation in its expression could alter glutamate homeostasis. In addition, a reduction in xCT levels in the hippocampus can also contribute to altered extracellular glutamate levels (De Bundel et al. 2011).

In this work, we observed that chronic exposure to As + F resulted in lower ALT activity (Fig. 4C). Previous work has shown that inhibition of glutamate transaminases by F exposure is associated with a reduction in total glutamate contents in the brain (Niu et al. 2009; Bartos et al. 2022). However, according to Andersen et al. (2021), ALT contribution to glutamate synthesis is less important than that of the glutamine–glutamate shuttle. Nonetheless, ALT activity could be important for glutamate buffering in astrocytes and ammonium recycling during the glutamine–glutamate shuttle. Erecinska et al. (1994) and Westergaard et al. (1993) showed that ALT activity is higher in astrocytes during glutamate degradation and alanine synthesis, favoring alanine release from astrocytes and its uptake by neurons. Moreover, the opposite cycling direction has been proposed to contribute to ammonium recycling (Schousboe et al. 2003). Although we cannot attribute the decreased ALT activity to a specific cell type or cellular location, alterations in its activity can clearly result in important alterations in glutamate metabolism and homeostasis.

The results obtained thus far suggest that As + F exposure alters glutamate homeostasis. Most likely associated with this, we observed that As or F exposure altered the proportion of AMPAR and NMDAR subunit expression in the cortex and hippocampus (Fig. 4A, B). Similar alterations in receptor expression have been observed previously for AMPAR and NMDAR expression after As or F exposure (Ramos-Chavez et al. 2015; Nelson-Mora et al. 2018; Sun et al. 2018; Yang et al. 2018); although at higher doses, both downregulated NR2A expression (Luo et al. 2009; Sun et al. 2018) (Fig. 4A).

NOR and LOR test results showed an important impairment in memory in mice exposed to As + F in tasks dependent on the cortex and hippocampus at P90 (Fig. 5). Other studies have shown that after exposure to As or F, memory impairment occurs in different hippocampal-dependent tasks, such as the water maze and location object recognition tests (Liu et al. 2014; Huo et al. 2015; Ramos-Chavez et al. 2015). However, some of these studies used higher doses of both elements. Here, we observed that As + F caused alterations in both tasks, in contrast to As or F exposure alone. Interestingly, in As-treated animals, the impairment seen in the cortex-dependent task could be associated with the higher accumulation of As in this brain region, as compared to the hippocampus. Moreover, memory impairment in this task occurred along alterations in AMPAR and NMDAR expression induced by the combination of As + F.

An increase in NR2A has been associated with memory impairment in different behavioral tasks, including object recognition (Cui et al. 2013). Additionally, reduction and ablation of the NR2B subunit in the forebrain result in impaired LTP and impaired performance in different spatial and nonspatial memory tasks (Clayton et al. 2002; von Engelhardt et al. 2008). The change in the NR2A/NR2B ratio, as a result of altered subunit expression, modifies the threshold for bidirectional synaptic plasticity (LTP or LTD) and influences spatial learning behavior (Bach et al. 1995; Zeng et al. 2001). The recruitment of AMPAR, especially GluA1-containing receptors to the synaptic area by electrophysiological-induced activity, results in the maturation and functional activation of the synapse as well as of its potentiation (Kessels and Malinow 2009). The reduction in the expression levels of receptors in the brain could result in improper postsynaptic activation, signal transduction, and memory codification.

Alterations in brain glutamate metabolism and availability as a result of redox imbalance during chronic exposure to As + F could lead to altered expression of glutamate receptors and learning and memory impairments as those observed in exposed subjects. Nevertheless, additional investigation regarding the activity of enzymes involved in the glutamine–glutamate cycle, extracellular glutamate levels, and glutamate vesicle release activity could better clarify the impact of these alterations on glutamate neurotransmitter availability. Moreover, electrophysiological evaluations could help explain the impact of As + F on glutamatergic synapses. It will also be interesting to evaluate whether these changes in glutamate receptor expression could also result in poor dendritic spine maturation (Haas et al. 2006) and altered brain interconnections.

Finally, this study, which involved mice exposed to biologically relevant levels of As and F, provides new insights into the alteration in transsulfuration pathway as a possible mechanism of xenobiotic-induced neurotoxicity, considering its involvement in H_2_S and cysteine production and their function in the brain. Future studies should explore the inhibition of the transsulfuration pathway by other xenobiotics in the CNS.

## Conclusion

Male mouse offspring exposed to As + F in combination through drinking water, starting at gestation, showed memory impairments at P90. These behavioral observations were associated with a downregulation of GSH levels and transsulfuration pathway activity and with a decrease in the levels of glutamate transporters as well as with a reduction in de novo synthesis of glutamate, suggesting an altered glutamate availability. Additionally, we observed changes in the proportion of NMDA glutamate receptor subunits, which may add to an imbalance in excitatory neurotransmission.

## Supplementary Information

Below is the link to the electronic supplementary material.

Supplementary file1 Supplementary Fig. 1 Schematic of behavioral testing for NOR and LOR. A) Representation of the squared arena used during open field and recognition tasks (NOR and LOR). B) Habituation to the arena was conducted for 10 min for three consecutive days without exposure to objects. C) Diagram and object placement for NOR and LOR. For the NOR task, two identical objects (A1 and A2, Lego pyramids) were used during acquisition on Day 4. The left object was the same during the test phase on Day 5 as in Day 4 (Lego pyramids) and is referred to as the “familiar object” (FO). The right object on Day 5 was a new figure (Lego tower) called the “novel object” (NO). For the LOR task, on Day 6, the object configuration used the day before was employed as a second acquisition trial to reduce novelty (L1, pyramid, and L2, tower). During the test phase on Day 7, the object referred to as “L2” (tower) during acquisition was displaced to a novel location (NL). In contrast, the object L1 (pyramid) used during acquisition was kept in the same place in the test phase, a familiar location (FL). D) Photographs of the objects employed during the recognition tasks (PDF 3843 KB)

Supplementary file2 Supplementary Fig. 2 CBS and CSE protein expression levels in the cortex (A) and hippocampus (B). Bars represent means ± SEMs of densitometric determination of Western blot images normalized against total protein stain as loading control and expressed respect to controls. Data were analyzed by two-way ANOVA followed by Tukey’s post hoc analysis. P <0.05. Representative image of PbS spots formed during the activity assay (C) (PDF 70 KB)

Supplementary file3 Supplementary Fig. 3 Behavioral analysis of the open field task at P90. Total traveled distance (A); rearing frequency (B) and % time spent in the center of the arena (C). Bars represent the mean ± SEM values, n=7 Data were analyzed by two-way ANOVA followed by Tukey’s post hoc analysis, *P<0.05 (PDF 37 KB)
