# Supplementary figures and images for: Chronic exposure to inorganic arsenic and fluoride induces redox imbalance, inhibits the transsulfuration pathway, and alters glutamate receptor expression in the brain, resulting in memory impairment in adult male mouse offspring
Source: Arch Toxicol. 2023 Jul 23;97(9):2371–83. doi: 10.1007/s00204-023-03556-7 (PMC10404204; doi:10.1007/s00204-023-03556-7)

**A**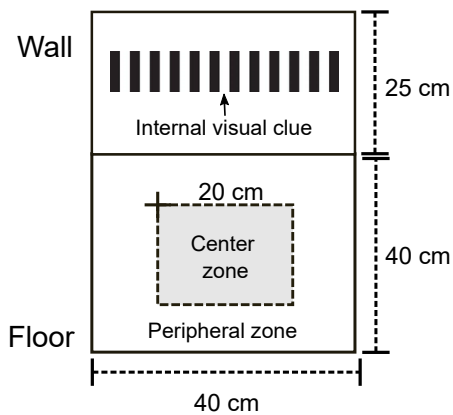**B**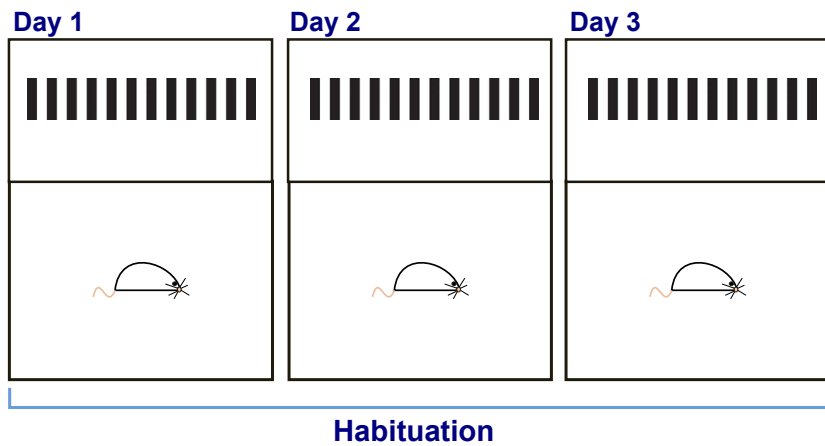**C**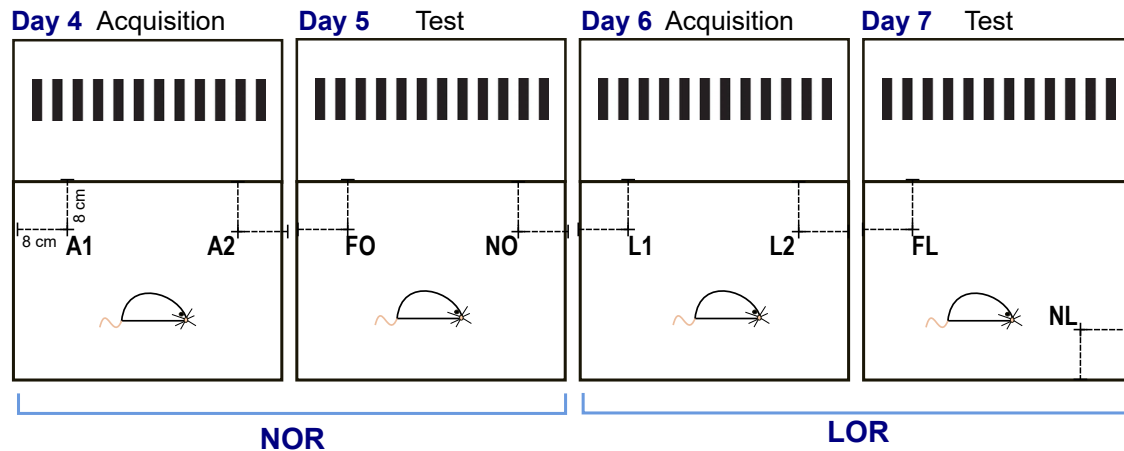**D**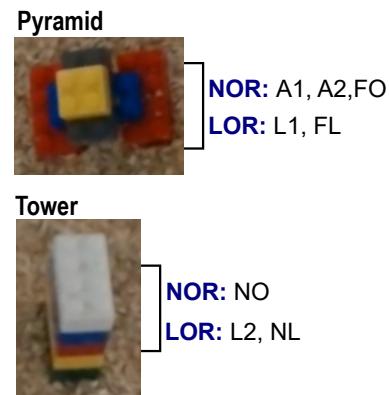

Supplement: Supplementary file 1 — Supplementary file1 Supplementary Fig. 1 Schematic of behavioral testing for NOR and LOR. A) Representation of the squared arena used during open field and recognition tasks (NOR and LOR). B) Habituation to the arena was conducted for 10 min for three consecutive days without exposure to objects. C) Diagram and object placement for NOR and LOR. For the NOR task, two identical objects (A1 and A2, Lego pyramids) were used during acquisition on Day 4. The left object was the same during the test phase on Day 5 as in Day 4 (Lego pyramids) and is referred to as the “familiar object” (FO). The right object on Day 5 was a new figure (Lego tower) called the “novel object” (NO). For the LOR task, on Day 6, the object configuration used the day before was employed as a second acquisition trial to reduce novelty (L1, pyramid, and L2, tower). During the test phase on Day 7, the object referred to as “L2” (tower) during acquisition was displaced to a novel location (NL). In contrast, the object L1 (pyramid) used during acquisition was kept in the same place in the test phase, a familiar location (FL). D) Photographs of the objects employed during the recognition tasks (PDF 3843 KB) [file 204_2023_3556_MOESM1_ESM.pdf]

**A****CORTEX**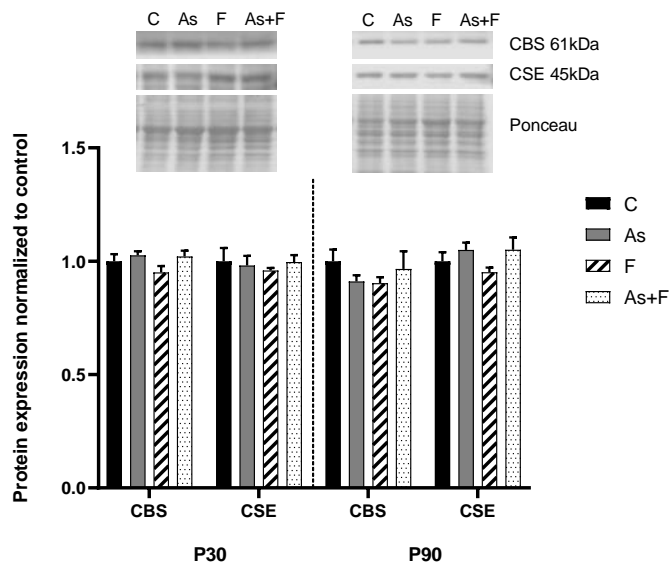**B****HIPPOCAMPUS**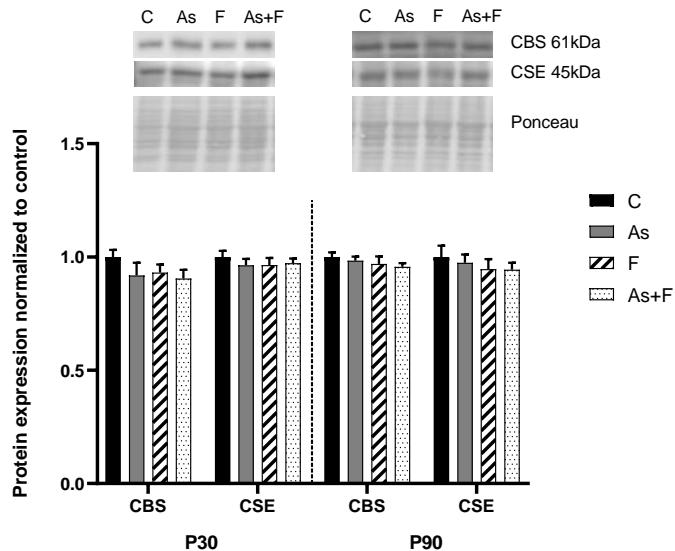**C**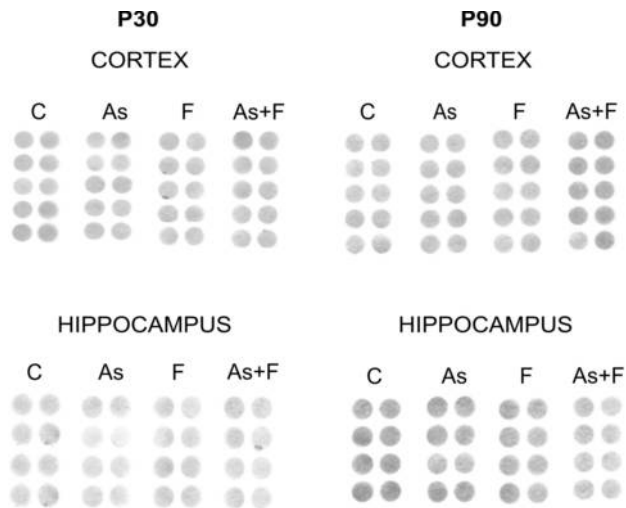

Supplement: Supplementary file 2 — Supplementary file2 Supplementary Fig. 2 CBS and CSE protein expression levels in the cortex (A) and hippocampus (B). Bars represent means ± SEMs of densitometric determination of Western blot images normalized against total protein stain as loading control and expressed respect to controls. Data were analyzed by two-way ANOVA followed by Tukey’s post hoc analysis. P <0.05. Representative image of PbS spots formed during the activity assay (C) (PDF 70 KB) [file 204_2023_3556_MOESM2_ESM.pdf]

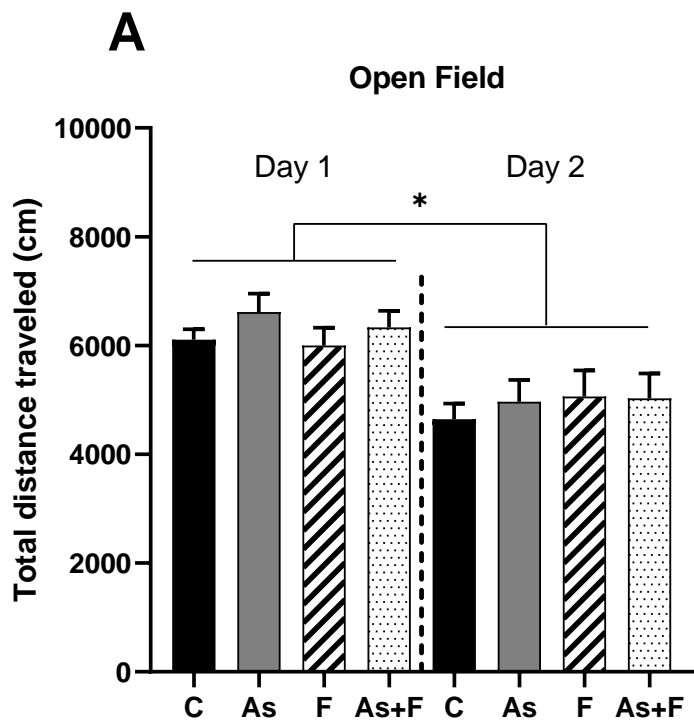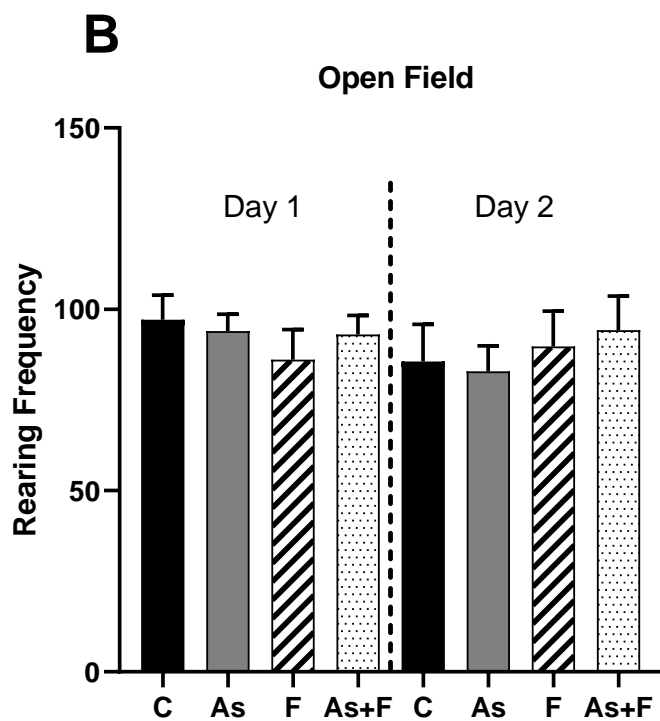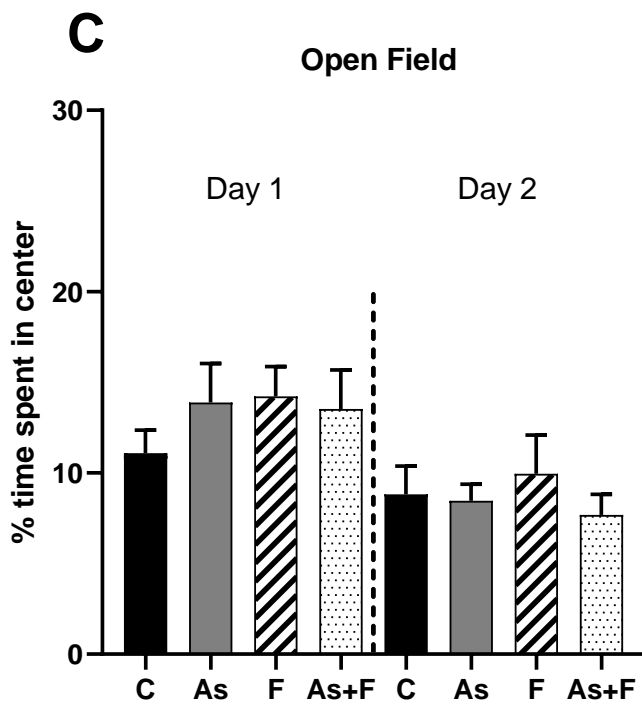

Supplement: Supplementary file 3 — Supplementary file3 Supplementary Fig. 3 Behavioral analysis of the open field task at P90. Total traveled distance (A); rearing frequency (B) and % time spent in the center of the arena (C). Bars represent the mean ± SEM values, n=7 Data were analyzed by two-way ANOVA followed by Tukey’s post hoc analysis, *P<0.05 (PDF 37 KB) [file 204_2023_3556_MOESM3_ESM.pdf]
